# Estimation of Soil Erosion Dynamics in the Koshi Basin Using GIS and Remote Sensing to Assess Priority Areas for Conservation

**DOI:** 10.1371/journal.pone.0150494

**Published:** 2016-03-10

**Authors:** Kabir Uddin, M. S. R. Murthy, Shahriar M. Wahid, Mir A. Matin

**Affiliations:** International Centre for Integrated Mountain Development, GPO Box 3226, Kathmandu, Nepal; University of Calgary, CANADA

## Abstract

High levels of water-induced erosion in the transboundary Himalayan river basins are contributing to substantial changes in basin hydrology and inundation. Basin-wide information on erosion dynamics is needed for conservation planning, but field-based studies are limited. This study used remote sensing (RS) data and a geographic information system (GIS) to estimate the spatial distribution of soil erosion across the entire Koshi basin, to identify changes between 1990 and 2010, and to develop a conservation priority map. The revised universal soil loss equation (RUSLE) was used in an ArcGIS environment with rainfall erosivity, soil erodibility, slope length and steepness, cover-management, and support practice factors as primary parameters. The estimated annual erosion from the basin was around 40 million tonnes (40 million tonnes in 1990 and 42 million tonnes in 2010). The results were within the range of reported levels derived from isolated plot measurements and model estimates. Erosion risk was divided into eight classes from very low to extremely high and mapped to show the spatial pattern of soil erosion risk in the basin in 1990 and 2010. The erosion risk class remained unchanged between 1990 and 2010 in close to 87% of the study area, but increased over 9.0% of the area and decreased over 3.8%, indicating an overall worsening of the situation. Areas with a high and increasing risk of erosion were identified as priority areas for conservation. The study provides the first assessment of erosion dynamics at the basin level and provides a basis for identifying conservation priorities across the Koshi basin. The model has a good potential for application in similar river basins in the Himalayan region.

## Introduction

Land degradation, sedimentation, and ecological degradation tend to increase as a result of inappropriate land use and management practices [1]. Soil erosion is contributing to substantial changes in basin hydrology and inundation [2] in the transboundary Himalayan river basins, and the problems are compounded by social, economic, and political changes [3]. Water-induced erosion in the mountain and hill areas of these basins is very high [4, 5] as a result of the steep slopes [6] as well as terrace agricultural practices with poor management. The rivers in the region transport heavy loads of sediment [7, 8] which are deposited downstream, leading among others to the formation of islands in the Ganges and Brahmaputra delta [6, 9]. Soil erosion has been reported to affect crop production [10], and also leads to sedimentation in dams [5, 11, 12]. Information on the spatial distribution patterns and dynamic changes in erosion across the river basins is needed to develop plans and determine priorities for controlling soil erosion at the river basin level.

The Koshi basin extends from the Tibetan Plateau in China, through Nepal, to the Gangetic plains in India. It has a diverse topography, geology, and geomorphology, and a wide range of different land use practices, and is also strongly affected by soil erosion, sediment transport, and land degradation [13–15]. The land and water resources of the basin are at risk as a result of rapid population growth, deforestation, soil erosion, sediment deposition, and flooding [16, 17] and are not used as effectively as they could be to improve the livelihoods and socioeconomic conditions of the local people [18]. The distinct topography and land cover scenario of the basin means that there are three different erosion regimes: 1) the high mountains with steep to moderate slopes and predominant land cover of grass, snow, and glaciers; 2) the middle mountains with steep to moderate slopes and predominant land cover of forest and agriculture; and 3) the low hills and plains with predominant land cover of agriculture. Studies based on small-scale erosion assessments using field or model-based methods have reported high erosion rates in the middle mountains of Nepal, which includes the most susceptible part of the Koshi basin [19, 20].

High and intense erosion is one of the most distinctive characteristics of the Koshi basin. The high levels of erosion result in high levels of sedimentation which affect storage infrastructure (filling of dammed lakes), can destroy agricultural land, and contribute to downstream fluvial hazards. Although it is well known that the high level of erosion and sedimentation is primarily the result of the young, fragile, and tectonically active nature of the Himalayan mountains, little is known in detail about the contribution from different geologic/geomorphic units and landscapes. This poses challenges for planning, especially planning of water infrastructure such as hydropower and irrigation schemes, where knowledge of the potential sedimentation risk is paramount, and planning to reduce erosion risk. The main approach used in sustainable sediment management is to reduce levels of erosion, although directing sedimentation can also play a role. But in order to be able to control erosion effectively, it is first necessary to have information about its spatial and temporal distribution. Erosion control also has an important role to play in reducing flood risk in the flood plains of Nepal and Bihar, where siltation following floods is one of the major causes of loss of useful agricultural land. Small scale field studies can help in planning erosion control measures for small catchments, but spatial information on erosion dynamics and quantity at the river basin scale is needed to plan effective soil conservation and erosion control measures that address the problems of siltation along the major rivers and downstream in the flood plain areas.

It is important to identify the most sensitive areas for soil erosion in the Koshi basin, so that priority areas can be determined for conservation measures, but this is methodologically challenging. Soil erosion management strategies in the Koshi basin are constrained by the scarcity and fragmented nature of the available data. Few field measurements have been carried out using standardised protocols, and none over the whole basin, and there have been very few studies that analyse the spatial trends in erosion and the relationship to land use practices and rainfall regimes. Most studies on erosion in the Koshi basin have focused on individual plots or catchments in the middle mountains of the Nepal Himalayas because the topography, land use dynamics, and high spatial and temporal variability in rainfall lead to higher levels of erosion [5, 7]. Although a number of researchers have attempted to fill the gap in erosion data at various scales [5, 8, 21, 22], none have presented information on erosion patterns and dynamics for the entire basin. This paper aims to help fill this gap by describing a relatively simple method for estimating the spatial distribution and total value of soil erosion across the whole basin.

Soil erosion can be estimated using empirical or physically-based models. Empirical soil erosion models include the Universal Soil Loss Equation (USLE) [23], Chemical Runoff and Erosion from Agricultural Management Systems (CREAMS) [24], Agricultural Nonpoint Source model (AGNPS) [25], Revised Universal Soil Loss Equation (RUSLE) [26], and Modified Universal Soil Loss Equation (MUSLE) [27]. In theory, physically-based models have an advantage over empirical models because they can be combined with physically-based hydrological models. Fully distributed physical models such as Water Erosion Prediction Project (WEPP) and Agricultural Non-Point Source Model (AGNPS) perform better than equation-based models, but the cost of computation is high and they require a large amount of input data at high spatial resolution [20, 28]. Complete listings and descriptions of different soil erosion models can be found in [29]. The empirical RUSLE model remains the most popular tool for assessing water erosion hazards due to its modest data demands and easily comprehensible model structure, especially in developing countries where the possibilities for applying more complex models are often limited by a lack of adequate input data. In recent decades, RUSLE and its adapted versions [26, 30] have been applied worldwide in different regions and at different spatial scales. The RUSLE-GIS interface has several advantages in terms of easy updating, integration of spatially referenced data, and the facility to present the mapping results in different forms. A number of studies have shown good results using RUSLE together with GIS methods and RS data to model soil erosion (e.g. [31–33]).

There have been a number of model-based studies of soil erosion in small individual watersheds in the Nepal Himalayas. RUSLE has been used successfully to assess soil erosion in the Trijuga [34] and Kulekhani [35] watersheds. Satisfactory results have also been obtained using the Revised Morgan, Morgan, and Finney (RMMF) model in the Pakhribas [36] and Likkhu Khola valleys [37], and RUSLE in the Bagmati basin [20]. Quincey and others Quincey et al. [38] used the Limburg Soil Erosion Model [39] to estimate soil erosion in the Pokhare Khola watershed at mid elevations, and high and medium spatial resolution optical images were used with a GIS to assess erosion-prone areas in the Mustang watershed [40]. RUSLE and RMMF have also been applied to the Kalchi Khola watershed to predict soil loss rates and the spatial erosion pattern [20]. In the present study, we used the RUSLE model together with remote sensing (RS) data and GIS to make a basin-wide assessment of erosion dynamics in the Koshi river basin and determine priority areas for soil conservation and erosion prevention.

## Materials and Methods

The study did not require any specific permission for field sites because most of the analysis was carried out using remotely-sensed data.

### Study Area

**Fig 1:**
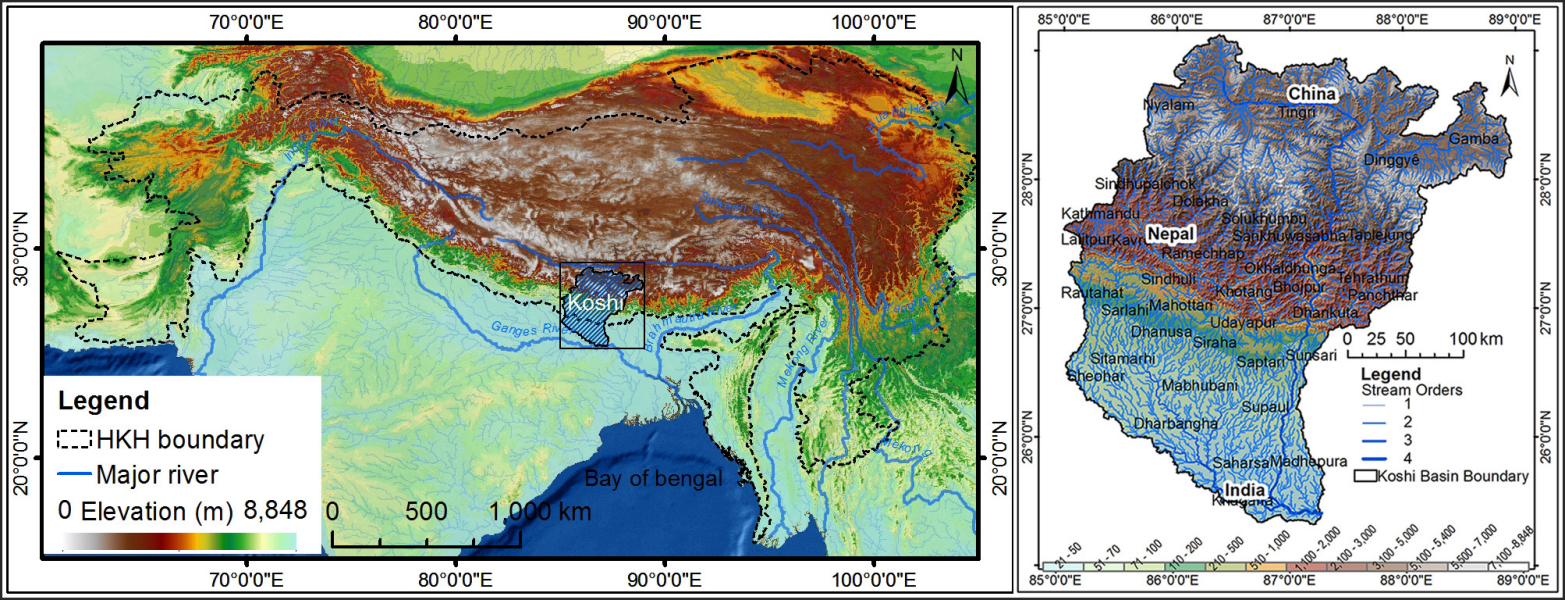
The Koshi basin.

The Koshi river basin lies between 85.02° and 88.95° E longitude and 25.33° and 29.14° N latitude, with a total area of 88,518 km^2^ (Fig 1) and encompasses the eastern highlands and lowland system of the Ganges river. The basin extends from the Tibet Autonomous Region in China, through Nepal, to Bihar State in India, and has seven major sub-basins: the Tama Koshi, Arun, Dudh Koshi, Likhu, Tama, Sun Koshi, and Indrawati. The basin contains a rich biodiversity and is a source of valuable ecosystem services that sustain the lives and livelihoods of millions of people in China, India, and Nepal [41] The regulating and support services include ground water recharge, flood control, and carbon sequestration, and contribute to both regional and global climate regulation.

The basin has five distinct landscapes: the Tibetan plateau, high mountains, middle mountains, low mountains and hills, and plains or Terai. The digital elevation model (DEM) from the Shuttle Radar Topographic Mission (SRTM) shows an elevation range from 21 to 8,848m [42], and slopes ranging from 0 to 88.76 degrees. About 15% of the basin area has a slope of more than 30 degrees. The climate in the northern and southern parts is different. Most of the basin is characterised by heavy precipitation during the monsoon season (June to September) when more than 80% of annual precipitation [43] occurs, but the extreme north lies in the rain shadow plains and arid hill areas of Tibet AR. The maximum average annual precipitation in the basin is 3078 mm and the minimum 207 mm [44].

The average outflow of the Koshi river is estimated to be 47.2 km^3^/year [45]. The Koshi is a powerful river system with a history of shifting direction and causing widespread damage in both Nepal and India, which has earned it the name of the ‘Sorrow of Bihar’. Many families live in fear of the river bursting its banks, and flooding their homes and land. At the same time, the ecosystem goods and services from the Koshi basin have contributed greatly to people’s livelihoods and the local economy and the water resources are used for irrigation, fishing, watering animals, and religious rituals, as well as normal domestic purposes.

### Data Processes

#### Combining the Universal Soil Loss Equation and GIS

**Fig 2:**
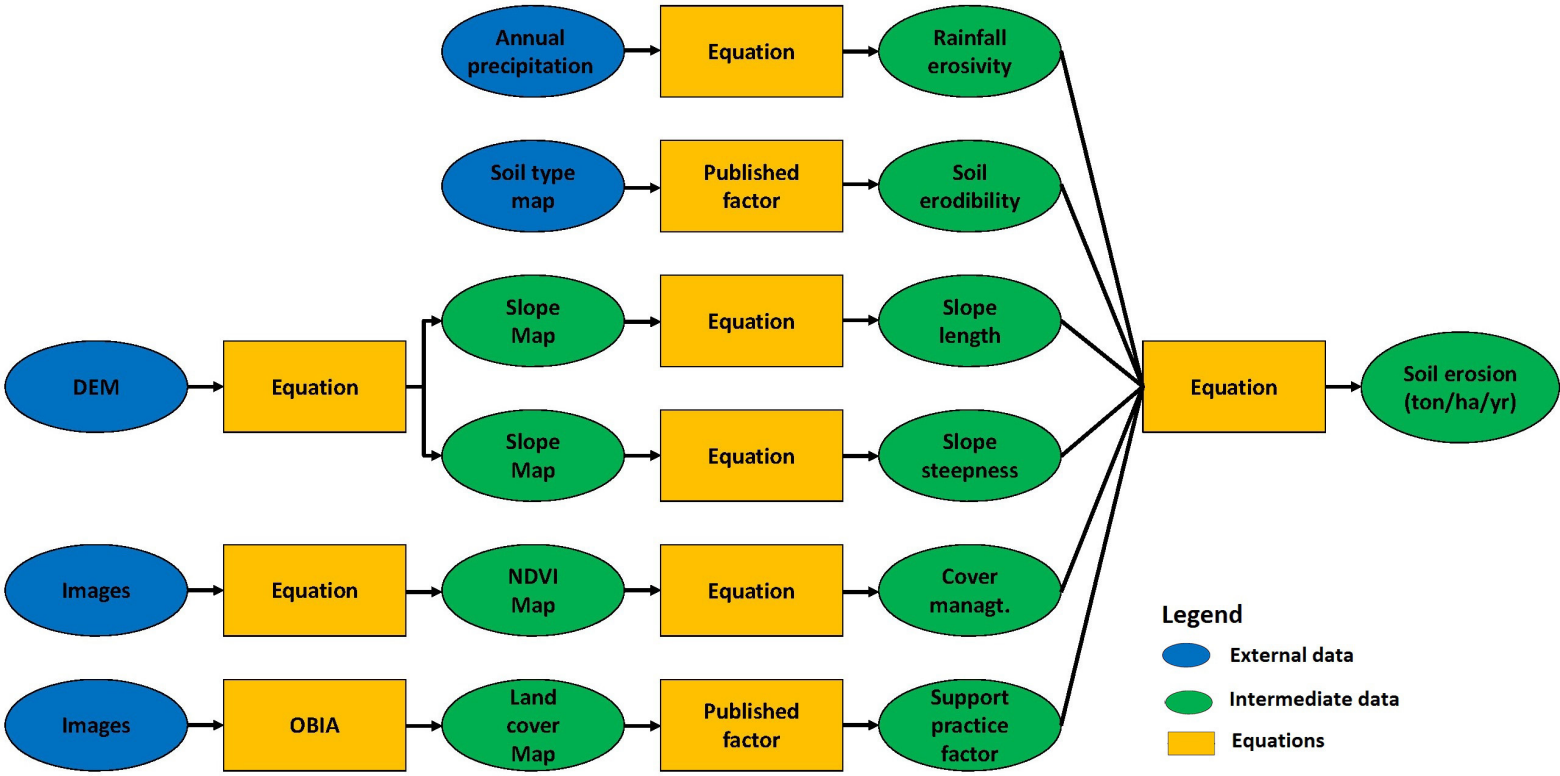
Flow chart for modelling soil erosion.

USLE and RUSLE are widely used to estimate rill erosion on overland flow areas. The equations use a combination of geophysical and land cover factors to estimate the likely annual soil loss from a unit of land. RUSLE was used to assess the spatial patterns of erosion risk in the study area. Recent advances in GIS and remote sensing technology have enabled a more accurate estimation of the factors used in the calculation [46, 47,48, 49]. Each of the factors was derived separately in raster data format and the erosion calculated using the map algebra functions. Fig 2 shows the framework for the RUSLE model calculation.

RUSLE is expressed as given in [23]:

A=R×K×L×S×C×P (1)

where, **A** is estimated average soil loss in t ha^-1^yr^-1^, **R** is the rainfall-erosivity factor, **K** is the soil erodibility index, **L** is the slope length factor (dimensionless), **S** is the slope steepness factor (dimensionless), **C** is the cover-management factor (dimensionless), and **P** is the supporting practices factor (dimensionless).

The RUSLE parameters were calculated using separate equations with input generated from satellite images and a DEM. The input data, their sources, and the equations used are listed in Table 1. The equations available in the literature for calculating the factors were tested iteratively and the optimal equations chosen based on their suitability for use with the data available and ability to produce estimates comparable to published field-based erosion measurements. The calculation of the individual factors is described in more detail in the next sections.

**Table 1:** Input data, sources, and equations used to calculate the RUSLE factors.

Factor	Input data	Data source	Equation used
Rainfall erosivity factor (R)	Precipitation (ESRI grids, 10 arc-minutes)	World climate precipitation data [50]	R = 0.0483*P1.610 (where P = annual precipitation (mm))
Soil erodibility factor (K)	Soil maps from Nepal, India, and FAO		Literature review
Slope length factor (L)	SRTM 90 m digital elevation data	[42]	L = (λ/22.13)^m^ where λ is the field slope length (m), and m assumes a value between 0.2 and 0.5 [51]
Slope steepness factor (S)	SRTM 90m digital elevation data	[42]	S = (0.43 + 0.30 s + 0.043 s2)/6.613 [23]
Land cover management factor (C)	NDVI from Landsat TM and ETM+	[52]	C = 0.431− 0.805*NDVI [53]
Support practice factor (P)	Land cover map	ICIMOD [54]	Literature review

#### Rainfall erosivity factor (R)

Annual rainfall erosivity is the total rainfall erosivity within a year. The rainfall erosivity factor (R) describes the erosivity of rainfall at a particular location based on the rainfall amount and intensity. This is an important parameter for soil erosion risk assessment under future land use and climate change [55]. A formula based on monthly rainfall proposed by McGarigal 2002 [56] was used with WorldClim precipitation data to calculate the R factor, which is expressed as:

R=0.0483*P1.610 (2)

where **P** = annual precipitation (mm).

**Fig 3:**
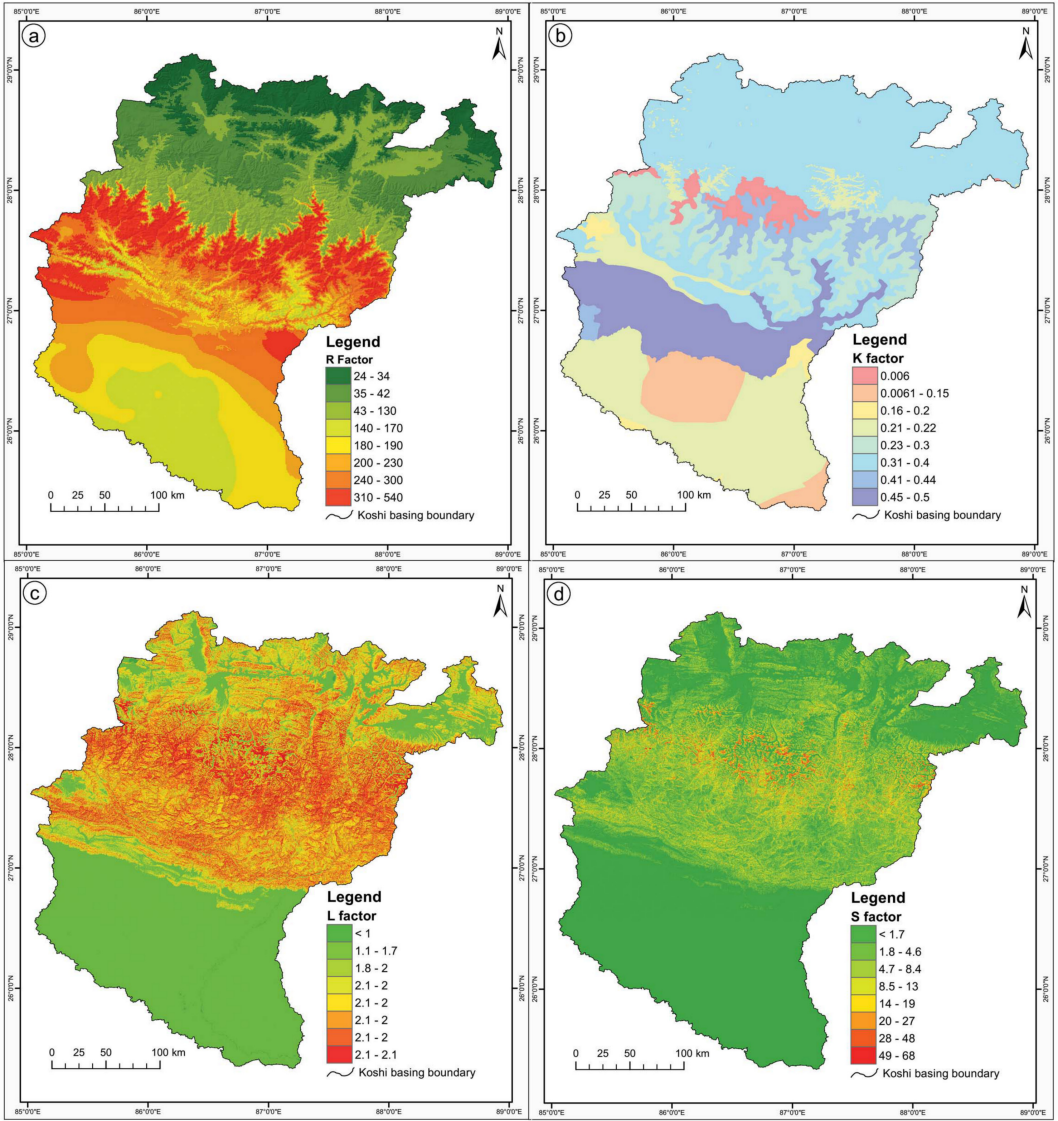
**Spatial distribution of four of the factors used in RUSLE: (a) rainfall-erosivity factor, (b) soil erodibility factor, (c) slope length factor, and (d) slope steepness factor.**

Fig 3A shows the rainfall erosivity factor map derived for the study area.

#### Soil erodibility factor (K)

The soil erodibility factor (K) is a quantitative description of the inherent erodibility of a particular soil type; it is a measure of the susceptibility of soil particles to detachment and transport by rainfall and runoff [57]. The main soil properties influencing the K factor are soil texture, organic matter, soil structure, and permeability of the soil profile. For a particular soil, the soil erodibility factor is the rate of erosion per unit erosion index from a standard plot. In this study, K values at soil order level were computed from the published literature on mountain areas [5, 7]. The erodibility of various soil types in the Koshi basin is given in Table 2. Fig 3B shows the spatial distribution of the soil erodibility factor in the study area.

**Table 2:** Erodibility factors for different soil classes in the Koshi basin.

Soil type	Erodibility factor (K-factor)
Udalfs(alfisols) Orthents(entisols)	0.10
Orthents(E) Aquepts(incepti)	0.20
Aquepts(i) Ochrepts(inceptisols)	0.10
Orthents(entisols) Ochrepts(inceptisols)	0.15
Orthents(e) Aquepts(i) Ochrepts(i)	0.01
Orthents (e)Fluvents(0.17)/entisols	0.20
Psamments(0.2)/entisols	0.15
Aquepts(i) Fluvents(e)	0.20
Aquepts(i) Ochrepts(i)	0.10
Orthents(e) Aquepts(i) Ochrepts(i)	0.10
Aquepts(i) Ustalfs(a)	0.10
Ustalfs(a) Ochrepts(i)	0.15
Aquepts(i) Ochrepts(i) Orthents(e)	0.15
Udalfs(a)	0.15
Aquepts(i) Ustalfs(a) Udalfs(a)	0.10
Orthents(e) Aquepts(i) Ustalfs(a)	0.15
Orthents (e)Tropepts	0.10
Ochrepts(i) Orthents(e) Udalfs(a)	0.15
Aqualfs(a) Fluvents(e) Aquepts(i)	0.50

#### Slope-length factor (L)

The SRTM DEM for the study area was used to calculate the slope length and slope steepness factors. The slope-length factor (L) represents the effect of slope length on erosion. It is the ratio of field soil loss to the corresponding soil loss from a 22.13 m length on the same soil type and gradient and is estimated using Eq (3).

$$L={\left(\lambda /22.13\right)}^{m}$$ (3)

Where, λ is the field slope length, and m has a value between 0.2 and 0.5.

Wischmeier and Smith [23] have described various ways of determining m for different slopes and these have been applied in the Indian subcontinent [7, 51]. In the present study, the value taken for m was based on the slope gradient and determined using the slope map as input (Table 3).

**Table 3:** Value of m for different slope gradients.

Slope gradient	Value of m
1%	0.2
1–3%	0.3
3–4.5%	0.4
4.5% or more	0.5

The field slope length λ was taken as the SRTM grid size (90 m); thus the slope length factor was calculated using Eq (4):

$$L={\left(\frac{90}{22.13}\right)}^{m}$$ (4)

Fig 3C shows the spatial distribution of the slope length factor in the study area.

#### Slope-steepness factor (S)

The slope-steepness factor (S) represents the effect of slope steepness on erosion. Soil loss increases more rapidly with slope steepness than it does with slope length. S is the ratio of soil loss from the field gradient to that from a 9% slope under otherwise identical conditions. The relationship of soil loss to gradient is influenced by the density of vegetation cover and soil particle size. The S factor is calculated using Eq (5) as described in [23]:

$$S=(0.43+0.30s+{0.043s}^{2})/6.613$$ (5)

where s is the slope in per cent.

Fig 3D shows the spatial distribution of the slope steepness factor in the study area.

#### Cover-management factor (C)

The cover-management factor C is used to reflect the effect of cropping and other management practices on erosion rates. Vegetation cover is the second most important factor next to topography controlling soil erosion risk [58]. The land cover intercepts rainfall, increases infiltration, and reduces rainfall energy. The C factor reflects the effect of surface cover, and practices that change the amount of surface cover, on erosion. In areas where land uses other than cropping dominate, as in the Himalayas, the C factor is normally assigned based on a simple assessment of vegetation cover, rather than close analysis of agricultural cropping patterns. We used the method proposed by De Jong [59] to generate the cover management factor (C) using the Normalized Difference Vegetation Index (NDVI) calculated from Landsat TM and ETM+ images from 1990 and 2010 (nine cloud free images for each time point, taken in November to January):

C=0.431−0.805*NDVI (6)

Where NDVI = near infrared (NIR)–red (R)/ near infrared (NIR) + red (R).

**Fig 4:**
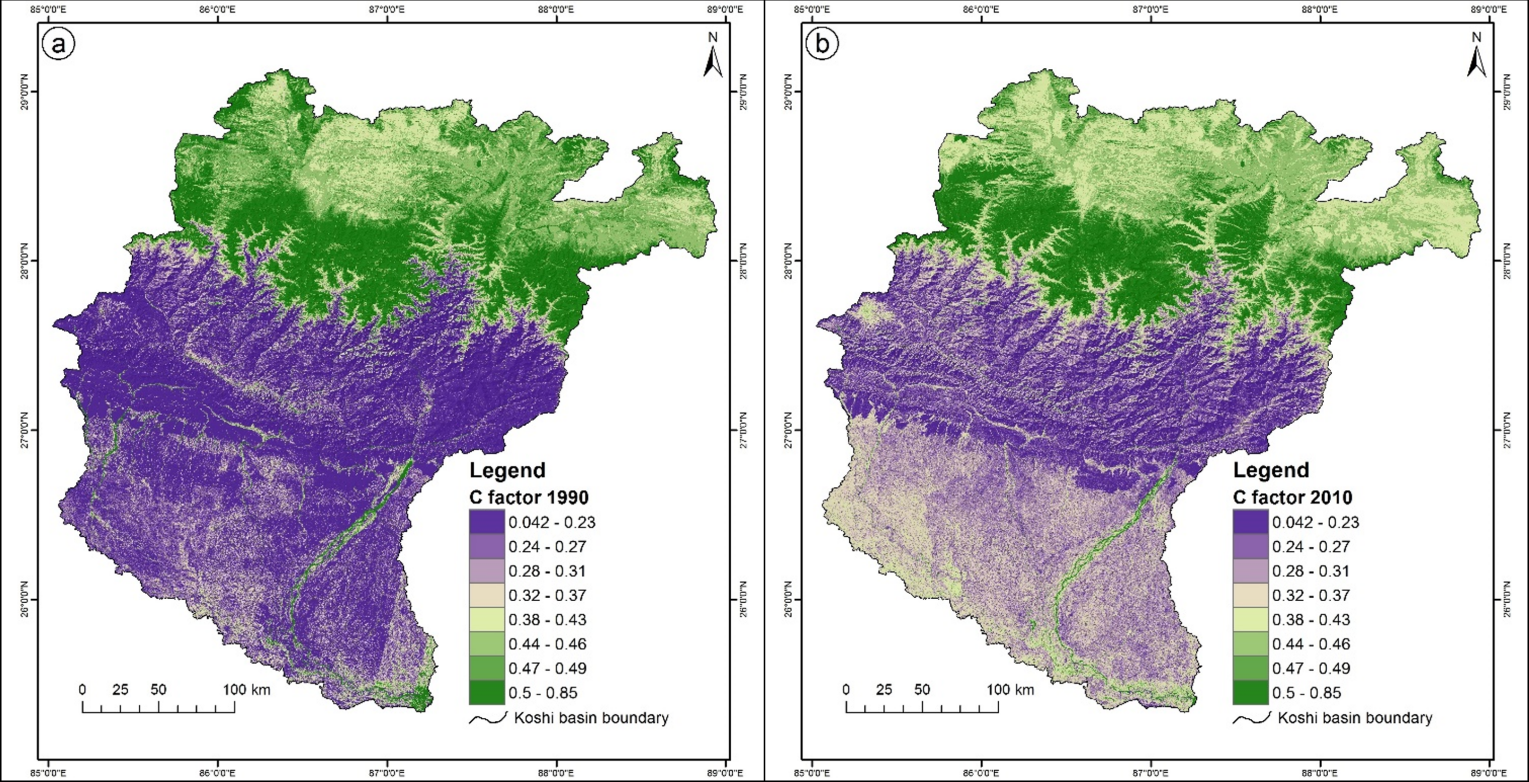
**Spatial distribution of the cover-management factor: (a) 1990, (b) 2010.**

Fig 4A and 4B show the spatial distribution of the cover-management factor in the study area in 1990 and 2010.

#### Support practice factor (P)

The support practice factor P reflects the impact of support practices such as contouring or strip cropping on the erosion rate. By definition, it is the ratio of soil loss with a specific practice to the corresponding loss with straight row ploughing up and down slope [53, 60]. Practices include all the different ways of using land, not simply agricultural practices, thus essentially the factor relates a particular type of land cover use to its erosion potential.

**Fig 5:**
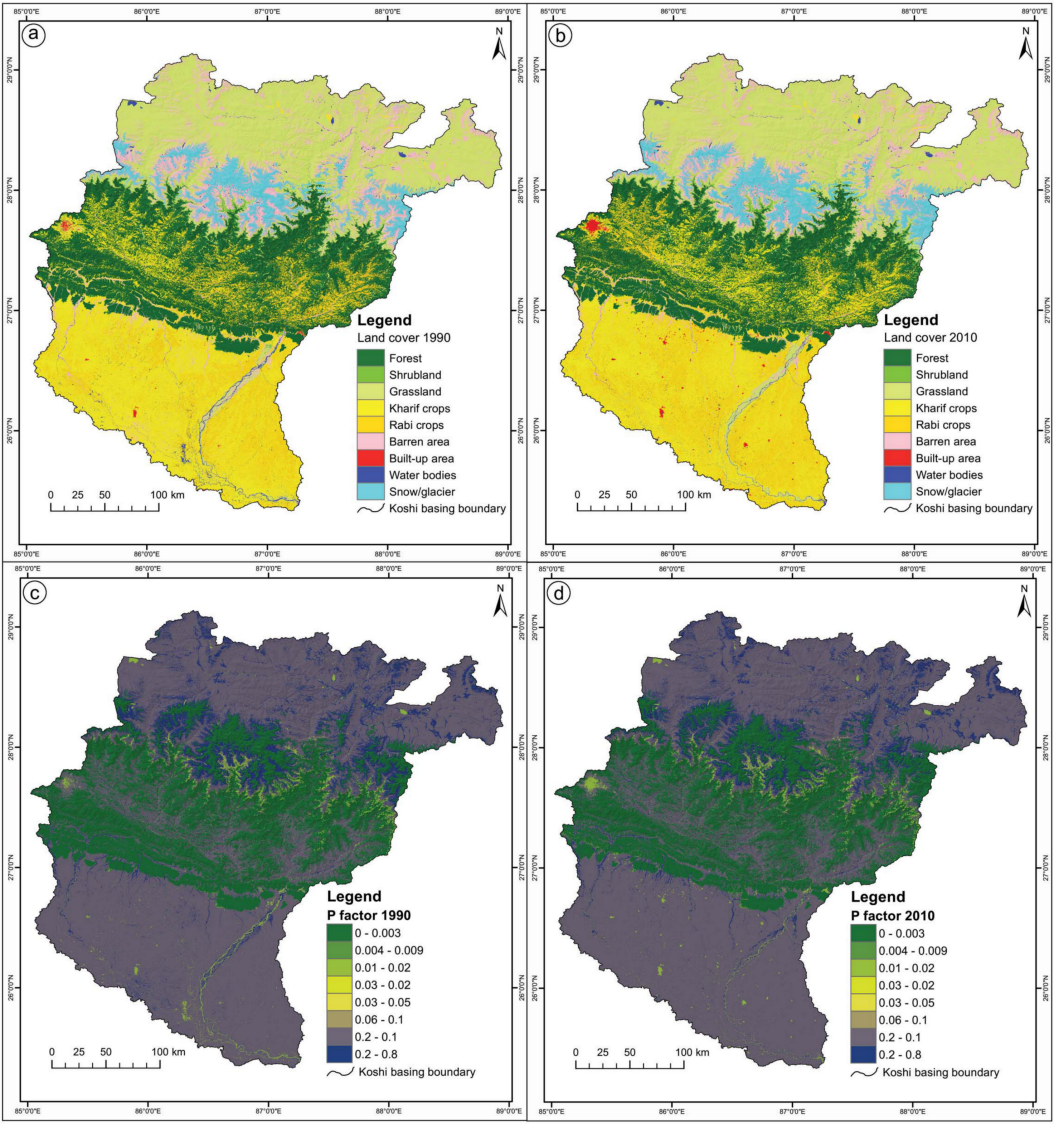
**Land cover map of the Koshi basin: (a) 1990, (b) 2010; spatial distribution of the support practice factor: (c) 1990, (d) 2010.**

As a first step, land cover maps for 1990 and 2010 were prepared from analysis of the Landsat TM and ETM+ images using object based image analysis [54, 61, 62]. The detailed methodology used to prepare the land cover maps is described in [54]. Briefly, eCognition Developer software was used to divide the image into segments that are similar in terms of selected attributes using indices like the Normalized Difference Vegetation Index (NDVI) and Normalized Difference Snow Index (NDSII) derived from spectral values of the image, together with a land water mask, and slope and texture information. The land cover maps for 1990 and 2010 are shown in Fig 5A and 5B.

Values for the support practice factor for particular types of land cover were taken from published sources [7, 53, 56, 63, 64] and linked with the land cover maps to generate maps of the spatial distribution of the support practice factor in the study area for 1990 and 2010 (Fig 5C and 5D).

## Results

**Fig 6:**
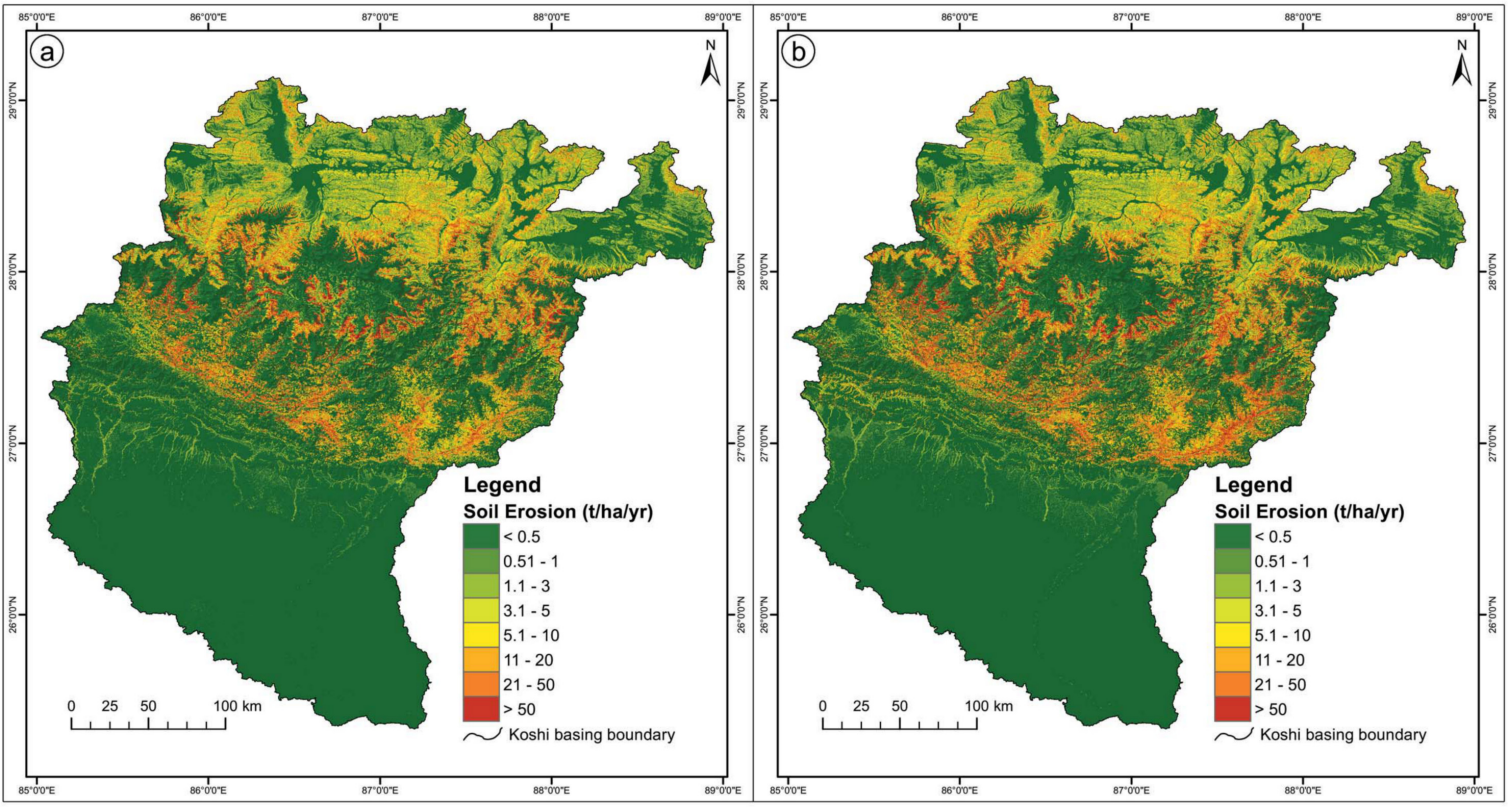
**Soil erosion risk map of the Koshi basin: (a) 1990, (b) 2010.**

Soil erosion risk maps were developed for the entire Koshi basin using RUSLE in conjunction with GIS and remote sensing data. The results are shown in Fig 6A (1990) and 6b (2010). The study area was divided into eight erosion risk classes, from very low to extremely high, based on the estimated erosion rates. The southern area of the basin was less erodible, and the central area highly erodible. The differences in erosion levels between the northern, central, and southern parts of the study area are mainly due to topography. The areas in the very low erosion class were mainly located at the lower elevations where the terrain is relatively flat. The estimated maximum per hectare average soil loss occurs at elevations between 1,000 and 2,000 masl and the minimum at elevations between 70 and 100 masl.

Table 4. shows the estimated soil loss from different land cover classes in 1990 and 2010. The maximum mean soil loss rate estimated was 22 t/ha/yr from barren land. The total soil loss from barren land was estimated to be 18 million t in 1990 and 15 million t in 2010. The total soil loss from agricultural land was estimated to be 10 million t in 1990 and 14 million t in 2010. The estimated total soil loss for the entire Koshi basin area was around 40 million tonnes (39 million tonnes in 1990 and 42 million tonnes in 2010).

**Table 4:** Land cover and estimated erosion rates in the Koshi Basin in 1990 and 2010.

Land cover	Land cover area (km^2^)		Annual soil loss (‘000 t)		Mean erosion rate (t/ha/yr)	
Year	1990	2010	1990	2010	1990	2010
Forest	20,032	19,827	601	991	0.3	0.5
Shrubland	679	670	231	261	3.4	3.9
Grassland	23,463	23,486	10,793	11,743	4.6	5
Agricultural land (kharif)	17,927	15,691	4,482	5,335	2.5	3.4
Agricultural land (rabi)	11,708	14,715	5,269	8,240	4.5	5.6
Barren land	8,245	7,081	18,057	15,437	21.9	21.8
Built-up area	99	268	0.5	2	0.05	0.08
Water bodies	793	572	56	11	0.71	0.19
Snow/glacier	4,595	5,235	5	5	0.01	0.01
Total	87,542	87,542	39,495	42,025		

Table 5 shows the transformation of area between erosion risk classes between 1990 and 2010 in the form of a change matrix. The area that remained constant in the different erosion classes is shown in bold in the diagonal cells. Close to 87% of the study area remained in the same erosion risk class. The proportion of the area at very low risk of erosion went down from 62.4% in 1990 to 60.5% in 2010, while the area at extremely high risk of erosion increased slightly from 1.8% to 1.9%. The erosion risk increased over 9.0% of the area, and decreased over 3.8%, indicating that overall the situation is worsening.

**Table 5:** Change matrix for erosion risk classes from 1990 to 2010 (%).

Soil erosion risk rank (t/ha/yr)	Very low (<0.5)	Low (0.5–1)	Low medium (1–2)	Medium (2–5)	High medium (5–10)	High (10–20)	Very high (20–50)	Extremely high (>50)	Total2010
Very low (<0.5)	**58.6**	0.8	0.2	0.2	0.1	0.1	0.2	0.3	60.6
Low (0.5–1)	2.0	**4.3**	0.3	0.0	0.0	0.0	0.0	0.0	6.6
Low medium (1–2)	0.3	0.5	**4.6**	0.3	0.0	0.0	0.0	0.0	5.7
Medium (2–5)	0.3	0.1	0.5	**6.7**	0.3	0.0	0.0	0.0	7.9
High medium (5–10)	0.3	0.1	0.1	0.8	**4.5**	0.3	0.0	0.0	6.1
High (10–20)	0.4	0.0	0.1	0.3	1.0	**3.7**	0.3	0.0	5.8
Very high (20–50)	0.4	0.0	0.0	0.1	0.2	1.0	**3.4**	0.2	5.3
Extremely high (>50)	0.2	0.0	0.0	0.0	0.0	0.0	0.3	**1.3**	1.9
Total 1990	62.5	5.8	5.8	8.4	6.2	5.2	4.3	1.8	**100.0**

It is important to determine priority areas for conservation to support decision making for soil and water conservation over the entire basin. In this study, we combined current erosion risk, actual estimated erosion, and changes in erosion risk to indicate priority areas to consider for conservation. A higher priority was assigned to areas with a high risk of erosion, estimated high level of soil loss, and increase in level of erosion. The multi-criteria decision rules for identifying conservation priorities were identified as described by others Wang et al. [65].

**Fig 7:**
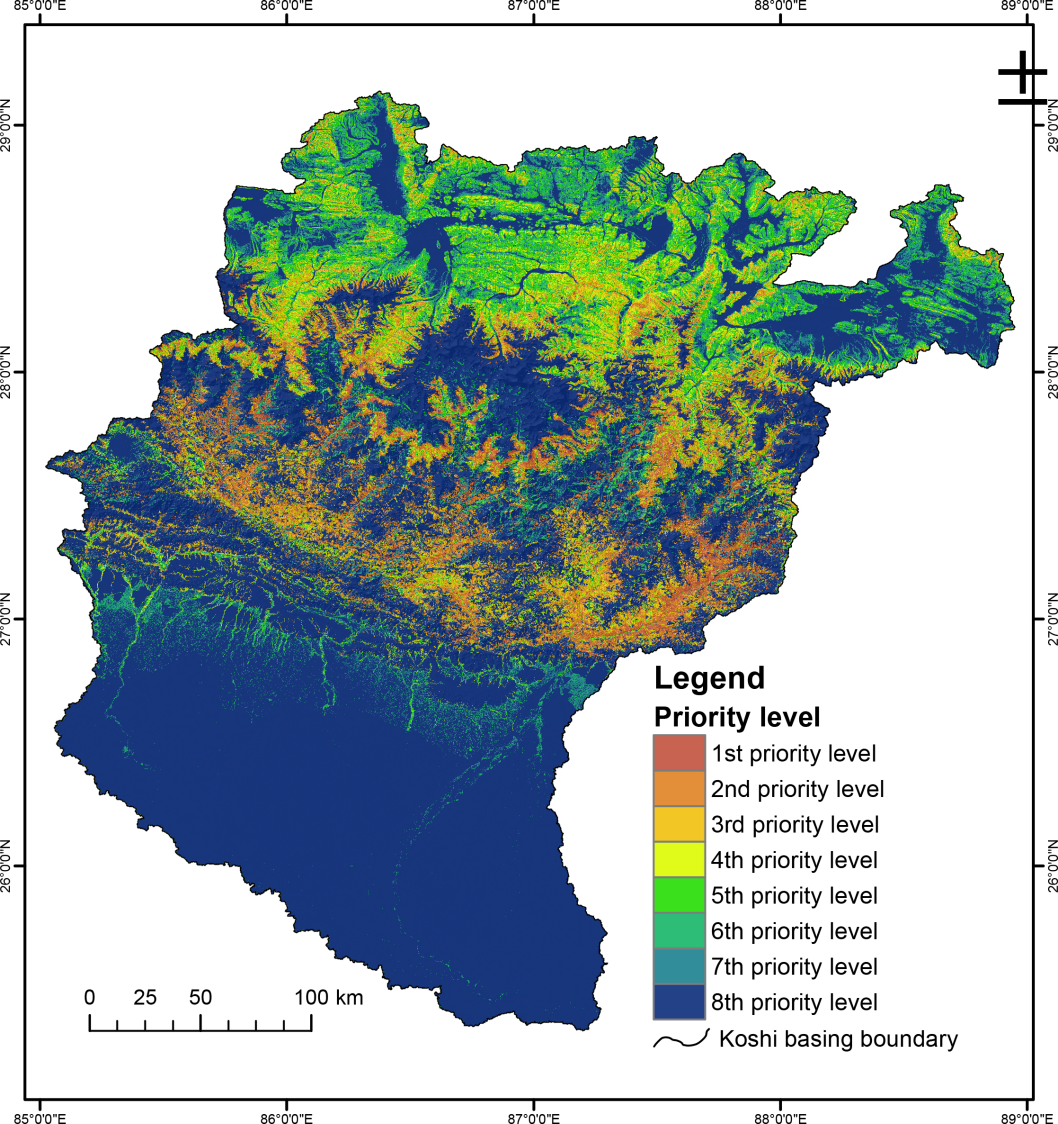
Priority areas for erosion control.

Fig 7 shows the conservation priority map obtained using this approach. The areas in the two top priority levels cover 7,758 km^2^ or close to 9% of the basin area and are mostly found in the central part of the basin at mid elevations. This area has the greatest intensity of agriculture, with high potential levels of erosion and an increase in erosion risk. The third and fourth levels cover 11% of the study area and represent areas with high but close to constant levels of erosion, while the lowest two levels (seven and eight) cover 66% of the basin area and represent areas with a low risk of erosion.

The conservation priority map and change matrix for erosion risk classes was also used to identify higher and lower priority districts for soil conservation following multicriteria decision rules. All high priority districts lie within Nepal. Dhankuta, Panchthar, and Tehrathum were identified as first priority districts, and Dolakha, Khotang, Okhaldhunga, Ramechhap, and Sindhupalchok as second priority (S4 Table).

## Discussion

The Koshi basin suffers from a very high level of erosion, which not only affects the land but also results in many negative impacts from sedimentation downstream. It is important to design and implement erosion control practices for the basin, but the area is large. To maximize their effectiveness, erosion control measures should be targeted at the most vulnerable areas, where the impact is likely to be greatest. But in order to do this, it is first necessary to understand the spatial pattern of erosion processes at the basin level. Field monitoring can provide useful information at a small catchment scale, but a very large number of such studies would be needed to cover the basin. Although a number of catchment level studies have been carried out in the Koshi basin [5, 7, 63, 66], there are no basin wide spatial data available; and there have been no basin-wide studies of erosion or erosion dynamics that can be used to determine priority areas for conservation activities. The study described here used a modelling approach–the RUSLE based method–to develop a detailed spatial assessment of the distribution of erosion risk across the entire Koshi basin using remotely-sensed data and automated analysis of land cover and slope gradient. This is the first time that such an approach has been used to assess erosion risk across an entire Himalayan river basin, and the methodology still has certain limitations, but it provides a useful means of identifying priority areas to consider for interventions to reduce soil erosion.

### Limitations and Future Possibilities

The method has some limitations and potential for improvement related to the factors that drive erosion in the RUSLE model, including rainfall, soil erodibility, slope length and steepness, and cover-management.

- Precipitation data from WorldClim were used together with annual rainfall-based equations suitable for hill areas to calculate the rainfall erosion factor. The number of weather stations in the Himalayan region is limited and the resolution of spatial precipitation data is low. Furthermore, this approach does not capture the distribution of heavy rainfall events, which are known to have a marked impact on soil erosion. The rainfall erosion potential is essentially determined by the product of total storm energy and maximum 30-min storm intensity. There are no detailed rainfall data available at sub-hourly intervals for the basin, but in future, hourly weather station data could be used to improve the estimates.
- The soil erodibility factor K was weighted at soil order level using published results [5, 66]. Better estimates could be made if more information can be obtained on soil texture and organic carbon.
- Several equations are available for estimating slope length factor from a digital elevation model. Most of these were found to overestimate erosion. The present study used the equation that gave the best estimate compared to the published literature. However, the slope length factor is one of the most important variables for erosion estimation and should be calibrated over the study area to increase the reliability of the quantitative estimates.
- Clouds obscure satellite images throughout much of the year and especially in the rainy season, thus the estimation of vegetation cover (NDVI) used in the calculation of the cover-management factor was restricted to the winter months. However, using spatio-temporal data fusion methods that integrate Landsat and MODIS data, for example the Spatio-Temporal Image Fusion Model (STI-FM)+ [67], would offer a way of compensating for this problem.
- Finally, comparison of two different time points was used to identify the change in erosion rates over time. This is a useful input for identifying sites to be investigated as a priority, but although changes can be related to changes in land use, cropping patterns, and other human controlled factors, they can also result from changes in precipitation, which can vary from locality to locality. In future studies, additional time points would help identify real trends; with further analysis to identify the main driver of the change.

It would be useful to assess the accuracy of the soil erosion estimates from the models using ground observations. It was not possible to validate the estimates and analyse error and bias by comparing model estimates with field-based measurements over a set of sites because there have been very few field-based studies in the basin. However, the results were compared with the estimated erosion levels of different land cover classes derived from published field data using plot level erosion measurements [5, 66, 68] and with other model-based results [37, 69, 70], mostly pertaining to mid and high hill areas in Nepal with similar characteristics to the Koshi basin. The RUSLE derived mean erosion rates for different types of land cover were within the range given by other authors (S1, S2 and S3 Tables) and the RUSLE models were relatively successful in predicting the relative pattern of soil loss. However, the mid hills of Nepal are extremely heterogeneous in terms of rainfall distribution, topography, soil, and cultural practices and this leads to a high variation in erosion levels. One-to-one comparison of the estimates over a set of sites is essential for proper validation and refinement of the model. In the future, such studies could be undertaken in the course of investigations of areas suggested for conservation activities, and an iterative process used to refine the model and improve recommendations.

Detailed ground-based studies would also be useful for testing other models. The RUSLE method has been reported to overestimate erosion in high terrain. The Rich Mesic Forest (RMF) and Mesic Forest (MF) models have been reported to yield better estimates over hilly terrain but require extensive ground data and calibration. A holistic discussion is needed on the accuracy required in erosion estimates in order to plan appropriate model and ground measurements.

### Soil Conservation and Identification of Priority Areas

Notwithstanding the limitations, the method offers a potentially very useful approach for identifying those areas likely to be most vulnerable to erosion and those that are likely to pose less risk, although the absolute values for erosion rates and soil loss should be considered with care.

Eight levels were differentiated with increasing priority for conservation on the basis of their erosion potential and identified change. Essentially the levels imply the following approaches. Levels 1 and 2, with highest priority, should be managed with some urgency in future projects as erosion control regions, and appropriate conservation strategies investigated and implemented. Levels 3 and 4 indicate areas that require a smaller allocation of funds to control soil erosion. Finally, Levels 5 to 8 have low erosion potential and will only require erosion control if the risk level increases, for example as a result of changes in land use. In these areas, land use planning should be used to ensure that the erosion risk is not increased by inappropriate changes in land use and/or poor planning of new infrastructure such as roads.

Soil conservation represents a complex biophysical, social, and economic challenge. Soil erosion is linked both to environmental degradation and to inappropriate land use practices, and is strongly affected by land use and land cover change, for example clearing of forest land for agriculture and infrastructure development [11]. The great majority of erosion in the Himalayan region is water related. Although many factors influence water erosion, vegetation cover, slope gradient, and land use play the most important role [30, 65]. Thus conservation efforts need to focus on vegetation cover and land use.

Identification of priority areas for investigation is a first step to facilitate planning. Ground level studies are needed in the high priority areas to determine the actual level of erosion on the ground, and where interventions can potentially be most useful in reducing erosion rates. Such studies are being considered under the Koshi Basin Programme coordinated by the International Centre for Integrated Mountain Development (ICIMOD) and implemented together with a number of country partners including government departments and (I)NGOs. Land cover management approaches such as afforestation of degraded land, improving infiltration through construction of pits, gully plugging, crop management for vegetation cover, and many others [71, 72] can be implemented at the field, hillslope, or watershed scale and the results assessed as a guide for future planning. Voluntary approaches can help to increase awareness among farmers; it is important to identify the best options for farming practices to reduce soil loss from cultivated land and provide support for the implementation of appropriate measures.

## Conclusions

The results presented here show that it is possible to use a modelling approach–the RUSLE based method–to develop a detailed spatial assessment of the distribution of erosion risk across an entire basin using remotely-sensed data and automated analysis of land cover and slope gradient. The results represent a best alternative to field-based measurement, which is not a viable option at the basin level.

According to Zhang et al. [72], conservation priorities can provide a significant criterion for decision making by government agencies. The conservation priority levels identified in this study indicate areas that are likely to be most in need of measures to address soil erosion; it is hoped that identifying these areas across the whole basin will support the planning of future erosion conservation actions in the Koshi basin. The model can be applied to similar river basins in the Himalayan region following appropriate calibration and validation.

## Supporting Information

S1 Table Surface erosion rates calculated in runoff plot studies reported by various authors. (DOCX)

S2 Table Soil erosion rates from field plot measurements reported by various authors. (DOCX)

S3 Table Model-based soil erosion estimates reported by various authors. (DOCX)

S4 Table District-wise priority levels for soil conservation activities. (DOCX)
