# Supplementary material for: Estimation of Soil Erosion Dynamics in the Koshi Basin Using GIS and Remote Sensing to Assess Priority Areas for Conservation
Source: PLoS One. 2016 Mar 10;11(3):e0150494. doi: 10.1371/journal.pone.0150494 (PMC4786292; doi:10.1371/journal.pone.0150494)
Supplement: S1 Table — (DOCX) [file pone.0150494.s001.docx]

**Table S1: District-wise priority levels for soil conservation activities: Surface erosion rates calculated in runoff plot studies reported by various authors**

| **Location and Plot Data** | **Land Use** | **Erosion Rate** | | **Source** |
| --- | --- | --- | --- | --- |
|  |  | t ha^-1^ | period |  |
| Siwaliks: Chatra, east Nepal; south aspect, sandstone; period of measurement and number of plots not given | Various, forest to grazing | 7.0–36.8 | year | Chatra Res. Centre,  Laban 1978 |
| Siwaliks: Gagretal, near Surkhet, west Nepal; south aspect, sandstone, average slope 60%; period of measurement and number of plots not given | Severely degraded heavily grazed forest on intensively gulled badlands. | 200 | year | Sakya, pers. comm. cited in Laban 1978 |
| Middle Mountains: Banpale, Phewa watershed, near Pokhara, Central Nepal; south aspect, elevation 1405 m, grey phyllitic schist, soils 40–70 cm clay loam, moderately well drained; one 10 m^2^ bounded plot on each land use type, four individual measurements 29 June – 5 July 1978 | Fenced pasture  Unfenced grazing land. | 9–4  34.7 | year | Mulder 1978 |
| Middle Mountains: identical location to Mulder 1978; two 10 m^2^ bounded plots each land use type; x – value of surface soil given as 0.35; daily measurements 11 June – 15 Oct. 1979 | Protected pasture mixed with forest. |  | June–Oct | Impat 1981 |
|  | Overgrazed land | 9.85 |  |  |
| Middle Mountains: Tamagi, Phewa watershed, near Pokhara; northeast aspect, elevation 1800 m, well drained clay loam derived from grey schist and quartzite schist; one 10 m^2^ bounded plot, 11 composite measurements 1 July – 7 Oct. 1979 | Dense forest. | 0.43 | July–Oct | Impat 1981 |
| High Mountains: Namche Bazaar to Dingboche, Sagarmatha National Park; 35 unbounded plots with 0.5 m long collection troughs; elevations 3440–4412 m; weekly measurements Mar–Oct. 1984. | Heavily grazed pasture | g/trough  10.5–715.4 | Mar–Oct | Byers 1986 |
|  | Utilized forest (litter/moss layer intact) | 0–16.2 |  |  |
