# Supplementary material for: Estimation of Soil Erosion Dynamics in the Koshi Basin Using GIS and Remote Sensing to Assess Priority Areas for Conservation
Source: PLoS One. 2016 Mar 10;11(3):e0150494. doi: 10.1371/journal.pone.0150494 (PMC4786292; doi:10.1371/journal.pone.0150494)
Supplement: S2 Table — (DOCX) [file pone.0150494.s002.docx]

**Table S2: Soil erosion rates from field plot measurements reported by various authors**

| **Place** | **Annual precipitation** | **Elevation** | **Plot type / Erosion rate t ha^-1^ yr^-1^** | | | | | **Publication** |
| --- | --- | --- | --- | --- | --- | --- | --- | --- |
|  | **(mm)** | **(masl)** | **Agriculture** | **Pasture** | **Degraded forest/ shrubland** | **Forest** | **Micro–watershed** |  |
| High Himal Khumbu | 807–1071 | 3300–4415 |  | 2.22–16.93 |  | 0.25–4.87 (0.6) |  | Byers 1987 |
| Langtang |  | 3000–4900 |  | 0.43–2.95 |  |  |  | Watanabe 1994 |
| High mountains Bamti - Bhandara | 1000–2200 | 1995–2453 | 0.2–12.7 (5.8) | 0.4 | 1.4 | 1.4 | 2.08–29.85 | Ries 1993 |
| Dandapakhar | 3125 | 1730 |  | 0.4–18.7 |  |  |  | Schaffner 1987 |
| Bonch | 3661 |  |  | 3.7–66.6 |  |  |  | Schaffner 1987 |
| Middle mountains Pakribas | 1261 |  | 16.9–36.7 (32.9) |  |  |  |  | Sherchan & Chand 1991 |
| Jhikhu Khola | 1393 | 1230–1260 | 0.1–42 (12.3) |  |  |  |  | Carver & Nakarmi 1995 |
| Chyandanda | 2104 | 1385 | 53.9–104.8 |  |  |  |  | Maskey & Joshi 1991 |
| Chisapani | 2047 | 1940 | 0.2–0.6 (0.6) |  |  |  |  | Maskey & Joshi 1991 |
| Kathmandu |  |  |  |  |  | 8 |  | Laban 1978 |
| Kulekhani | 1387 | 1620–1800 | 0.3–3.98 |  |  |  | 0.2–0.3 | Upadhaya et al. 1991; DSC 1995a |
| Phewa | 3700 |  |  | 9–35 |  | 0.34 | 15.2–15.4 | Mulder 1978; DSC 1996 |
| Dailekh |  |  | 2.7 | 20 | 15 | 5 |  | Carson 1985 |
| Churia |  |  |  |  |  |  |  |  |
| Chatra |  |  |  | 36.8 | 7.8 |  |  | Laban 1978 |
| Lothar |  |  |  | 31.5–420 |  |  |  | Laban 1978 |
| Surkhet | 923 | 720 | 1.06–2.74 (1.87) |  |  |  |  | DSC 1995b |
| Nepal |  |  |  |  |  |  |  | LRMP |
| Likhu Khola |  |  | 2.7–8.2; 11 (uncultivated) | 0.05 |  |  |  | Shrestha 1997 |
| Kavre watershed |  |  |  |  |  |  | 14.39 | Maskey and Joshi 1991 |
| Kulekhani watershed |  |  |  |  |  |  | 3.01 | Upadhyaya et al 1991 |
