# Supplementary material for: Estimation of Soil Erosion Dynamics in the Koshi Basin Using GIS and Remote Sensing to Assess Priority Areas for Conservation
Source: PLoS One. 2016 Mar 10;11(3):e0150494. doi: 10.1371/journal.pone.0150494 (PMC4786292; doi:10.1371/journal.pone.0150494)
Supplement: S3 Table — (DOCX) [file pone.0150494.s003.docx]

Table S3: Model-based soil erosion estimates reported by various authors

| **Place** | **Erosion  t ha^–1^ yr^–1^** | **Model** | **Reference** |
| --- | --- | --- | --- |
| Kalchi kola watershed | 3 (agric) | MMF model (1984) | Manoj and Poudel 2010 |
| Pokhare Khola | 8.6 (without terracing) | LISEM model | Quincey et al. 2007 |
| Mahadevkola sw | 6.1–56.2 (rainfed crops)  1.6–19.8 (rangeland)  0.1–8.6 (degraded forest)  0.–0.4 (dense forest)  0.1–0.8 (irrigated rice) | MMF model (1984) |  |
| Lidder catchment,India | 1–36(max 64) | USLE model | Ashak et al. 2011 |
| Bagmati basin,Nepal | 1–10 (forest)  10–100 (ag)  10–100 (shrub) | RSULE model | Jha 2002 |
| Trijuga watershed,Nepal |  | USLE model | Saha 1996 |
| Kulekhani Watershed ,Nepal |  | RMMF model | Kharel 1999 |
| Himalayan watershed | 1–10 (forest)  58 (agric) | USLE/Morgan models | Jain et al. 2002 |
