# Supplementary material for: Estimation of Soil Erosion Dynamics in the Koshi Basin Using GIS and Remote Sensing to Assess Priority Areas for Conservation
Source: PLoS One. 2016 Mar 10;11(3):e0150494. doi: 10.1371/journal.pone.0150494 (PMC4786292; doi:10.1371/journal.pone.0150494)
Supplement: S4 Table — (DOCX) [file pone.0150494.s004.docx]

Table S4: District-wise priority levels for soil conservation activities

| **District** | **Priority level** |
| --- | --- |
| Dhankuta | 1 |
| Panchthar | 1 |
| Tehrathum | 1 |
| Dolakha | 2 |
| Khotang | 2 |
| Okhaldhunga | 2 |
| Ramechhap | 2 |
| Sindhupalchok | 2 |
| Dingri | 3 |
| Niolam | 3 |
| Taplejung | 3 |
| Dingji | 4 |
| Sankhuwasabha | 5 |
| Bhojpur | 5 |
| Solukhumbu | 5 |
| Bhaktapur | 7 |
| Gangba | 7 |
| Kathmandu | 7 |
| Kavre | 7 |
| Lalitpur | 7 |
| Makwanpur | 7 |
| Sindhuli | 7 |
| Sαjia | 7 |
| Udayapur | 7 |
| Dhanusa | 8 |
| Dharbangha | 8 |
| E-chamarpan | 8 |
| Khagaria | 8 |
| Mabhubani | 8 |
| Madhepura | 8 |
| Mahottari | 8 |
| Rautahat | 8 |
| Saharsa | 8 |
| Saptari | 8 |
| Sarlahi | 8 |
| Sheohar | 8 |
| Siraha | 8 |
| Sitamarhi | 8 |
| Sunsari | 8 |
| Supaul | 8 |
